# Cell-specific targeting by engineered M13 bacteriophage expressing VEGFR2 nanobody 

**DOI:** 10.22038/IJBMS.2018.26191.6432

**Published:** 2018-09

**Authors:** Farideh Ranjibar, Mahdi Habibi-Anbouhi, Fatemeh Kazemi-Lomedasht, Seyed Hamid Aghaee-Bakhtiyari, Ehsan Alirahimi, Mahdi Behdani

**Affiliations:** 1 Biotechnology Research Center, Venom & Biotherapeutics Molecules Lab, Pasteur Institute of Iran, Tehran, Iran; 2 National Cell Bank of Iran, Pasteur Institute of Iran, Tehran, Iran; 3 Biotechnology Research Center, Mashhad University of Medical Sciences, Mashhad, Iran; 4 Department of Medical Biotechnology, Mashhad University of Medical Sciences, Mashhad, Iran

**Keywords:** Bacteriophage, Nanobody, Receptor-mediated gene- transfer, Targeted-gene delivery VEGFR2

## Abstract

**Objective(s):**

Filamentous bacteriophage M13 was genetically engineered to specifically target mammalian cells for gene delivery purpose.

**Materials and Methods:**

A vascular endothelial growth factor receptor 2 (VEGFR2)-specific nanobody was genetically fused to the capsid gene III of M13 bacteriophage (pHEN4/3VGR19). A mammalian expression construct containing Cop-green fluorescent protein (Cop-GFP), as a reporter gene, was amplified by PCR and then sub-cloned in the pHEN4/3VGR19 phagemid. The resulting construct was transfected into 293KDR cell. The recombinant phage was extracted and confirmed and then transduced into VEGFR2 expressing cell (293KDR).

**Results:**

Seventy-two hr after transfection, green fluorescence was detected in 30% of the cells. About 1% of the cells which transduced by recombinant phages were able to express GFP.

**Conclusion:**

It is hoped that the results from this study will help to find potential vectors to improve the efficiency of gene delivery. Taken together, we conclude that this newly-introduced vector can be used in cancer researches.

## Introduction

The success of gene therapy requires the ability to effectively deliver therapeutic genes to target cells while sparing normal cells from vector-associated toxicity (1-3). Current methods of gene delivery system are based on viral and non-viral systems. Non-viral methods such as lipid based techniques, calcium phosphate or dendrimers, are non-infective methods but are not as effective as viral based techniques. Viral methods, such as adeno-associated, lenti- or retroviruses on the other hands, are highly effective in transducing the mammalian cells but are infective and considered potentially hazardous for human or animal. Bacteriophage vectors combine the best properties of both viral and non-viral vectors. Additionally, these type of vectors lack intrinsic tropism for mammalian cells and can be conveniently produced at high quantities in bacterial culture, making their production much simpler and more economical than other methods (4). Moreover, phage particles can be easily packaged by using cell free systems (5). 

Bacteriophage vectors can be modified and engineered to display foreign peptides, proteins and antibodies as a fusion partner, with their coat proteins such as pIII or pVIII for monovalent and polyvalent expression of the fusions, respectively (6). These modifications can be done to increase the tropism of phage toward a specific cell type. Genetic flexibility, the most significant advantage of filamentous phages, allow a wide variety of peptides, proteins and antibodies to be displayed on the phage surface, improving the phage’s ability to target cell surface receptors (2, 7). Phage-mediated gene transfer depends on ligands as well as the time and dose of transduction (8, 9). 

Previously, we have developed a vascular endothelial growth factor receptor 2 (VEGFR2)-specific nanobody capable of recognizing VEGFR2 on the cell surface (10) or intracellular (11) and inhibiting *in vitro* endothelial tube formation (10). In the present study, recombinant bacteriophage was engineered that express VEGFR2-specific nanobody on surface fused to its pIII gene. By using Cop-green fluorescent protein (Cop-GFP) as a reporter gene, we showed that our engineered bacteriophage is successful at transducing and internalization of VEGFR2-expressing cell. We also showed that transduction and internalization of our bacteriophage is strictly depended on the presence of our ligand on the surface of phage. 

## Materials and Methods

***Cell lines and cell culture***

HEK293T and 293KDR cell lines were purchased from Pasteur Institute of Iran (Tehran, Iran). The cells were cultured in Dulbecco’s modified Eagle’s medium (DMEM) supplemented with 10% FBS (fetal bovine serum), 100 unit/ml penicillin, 100 µg/ml streptomycin, 2 mM L-glutamine and incubated at 37 ^°^C in a humidified incubator in an atmosphere of 5% CO_2_. 293KDR is a stably transfected cell line overexpressing VEGFR2 (12).

***Construction of phagemid for mammalian-cell reporter gene***

**Figure 1:**
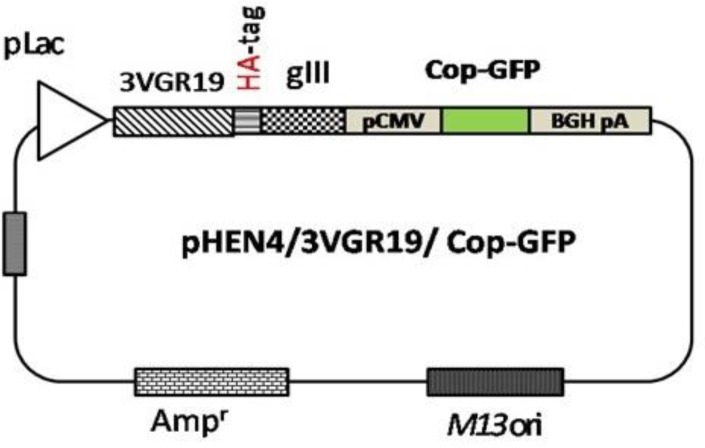
Schematic structure of pHEN4/3VGR19/copGFP plasmid

The final phagemid vector, designated pHEN4/ 3VGR19/Cop-GFP, was constructed. The overall structure of this construct is shown in Figure 1. Briefly, a GFP-expressing cassette was amplified by PCR from the pCMV/myc/ER/GFP plasmid as a template. PCR was performed with the following primers: pCMV (*EcoR *I) 5′-GTACCGAATTCACATTGATTATTG-3′, and BGH (*Nar *I) 5′-ACTGGGCGCCCGCCTCAGAAGCCATAGAG-3′. PCR products were cloned into the pHENE4/3VGR19 phagemid (10). The vector pHEN4/3VGR19/Cop-GFP was then generated by replacing the Cop-GFP sequence in the previous vector (13). The Cop-GFP fragment from the pCDH-CMV-MCS-EF1-cGFP-T2A-Puro plasmid was amplified using the following primers containing *Nhe *I and *Sac *I restriction sites, Cop-GFP (*Nhe *I), 5′-ACGGCTAGCGAGAGCGACGAGAGCG-3′, and Cop-GFP (*Sac *I) 5′-ACGGAGCTCGCGAGATCCGGTGGAG-3′). The resulting plasmid, was confirmed by colony-PCR, restriction digestion and DNA sequencing analysis. 

***Confirmation of Cop-GFP expression***

HEK293T and 293KDR cells were used to confirm Cop-GFP expression in the pHEN4/3VGR19/Cop-GFP phagemid. Cells were transfected using the standard calcium phosphate method. Cells were seeded in 6-well plate, and allowed to grow to 70-80% confluency. Three microgram of pHEN4/3VGR19/Cop-GFP and pCDH-CMV-MCS-EF1-cGFP-T2A-Puro (as control) plasmids were transferred into a sterile 15 ml conical centrifuge tube containing TE 1X, CaCl_2_ (2.5 M), 2x HBSS and buffered water. The resulting transfection mixture was mixed thoroughly and added to the wells in a drop-wise, plates were then incubated at 37 ^°^C. Forty-eight hr post-transfection, transfection efficacy of the reporter gene, Cop-GFP, was measured under fluorescence microscopy.

***Construction of M13 phage displaying VEGFR2-specific nanobody***

pHEN4/3VGR19/Cop-GFP was transformed into *Escherichia coli *TG1. An aliquot of bacterial suspension was incubated in 300 ml of 2xTY medium, supplemented with 4% (W/V) glucose and 100 µg/ml of ampicillin, at 37 ^°^C with shaking at 250 r/min until the OD_600_ was reached 0.5 (∼2-3 hr). The exponentially growing *E. coli *TG1 cells were then infected with 2×10^12^ VCSM13 helper phages for 30-60 min at 37 ^°^C. The infected cells were harvested by centrifugation at 4000 r/min for 15 min, pellets were re-suspended in 300 ml of 2xTY medium supplemented with 0.1% glucose, 100 µg/ml of ampicillin and 50 µg/ml of kanamycin followed by incubation overnight at 30 ^°^C with shaking at 250 r/min. Phage particles in the supernatant were precipitated with PEG/NaCl solution, followed by centrifugation at 8000 r/min. Phage titers were estimated by measuring the absorbance of the sample at 260 nm, according to the following empirical formula: phage ml^-1^ = OD_260_ × 100 × 22.14 × 10^10^.

***Analysis of recombinant phage particles***

Recombinant phage particles were confirmed using colony-PCR. Additionally, PCR amplification was used to verify the presence of pHEN4/3VGR19/Cop-GFP phagemid in the recombinant phage as a PCR template. To this end, *E. coli *TG1 cells were infected with recombinant and helper phages (as control), and colonies grown on the selective medium were investigated for the presence of the pHEN4/3VGR19/Cop-GFP phagemid using colony-PCR with the three sets of primers, including pCMV (*EcoR *I) and BGH (*Nar *I), Cop-GFP (*Nhe *I) and Cop-GFP (*Sac *I), and Rp and GIII primers, which recognizing the pHEN4 plasmid backbone, to confirm the presence of the pCMV promoter and BGH poly A tail, Cop-GFP gene, and the correct size of the construct, respectively. 

***In vitro*** ***transduction*** ***of phage particle ***

The ability of Cop-GFP phage particles to specifically target and transduce cells was evaluated using 293KDR cells expressing high levels of VEGFR2. A density of 5×10^4^ cells/well was seeded in 24-well plates. After 24 hr, cells were treated with helper phages, as negative control and recombinant phages at a density ranging from 10^7^ to 10^11^ cfu/ml. All cell cultures were incubated for 72 hr at 37 ^°^C in DMEM medium supplemented with 10% FBS prior to determination of Cop-GFP-positive cells. The transduction efficiency was measured as the percentage of total cells that were GFP positive using fluorescence microscopy analysis.

## Results

***Construction of recombinant phagemid vector***

Enzymatic digestion was used to confirm the cloning procedure of pHEN4/3VGR19/Cop-GFP. *Sac *I and *Nhe *I digestion yielded a fragment of approximately 755bp which confirmed the presence of the Cop-GFP cassette gene (data not shown).

***Recombinant phagemid transfection and reporter gene detection***

**Figure 2:**
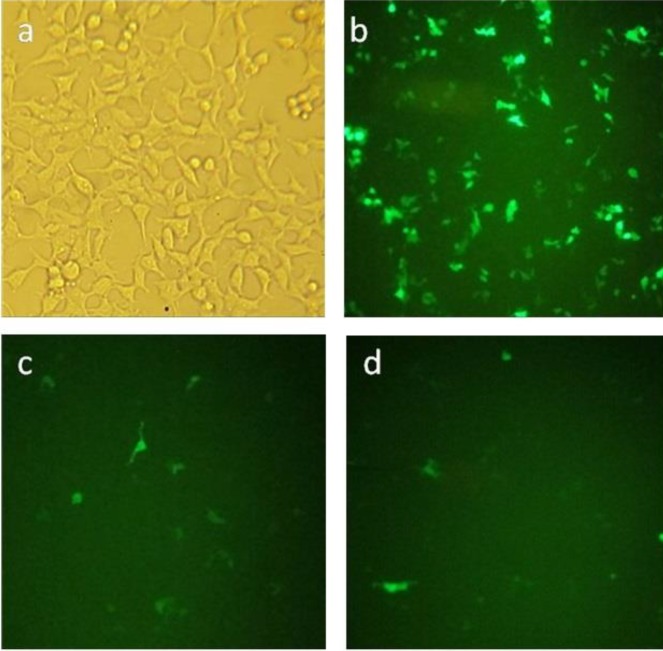
*In vitro* transfection of 293KDR and HEK293T cell lines with pHEN4/3VGR19/cop-GFP phagemid particles. (a) HEK293T cells; (b) 293KDR cell transfected with pCDH-CMV-MCS-EF1-GFP-T2A-Puro plasmid using the standard calcium phosphate method as a positive control; (c) Transfected 293KDR cells; (d) Transfected HEK293T cells

To verify whether the phagemid can be expressed in mammalian expression systems, pHEN4/3VGR19/Cop-GFP phagemid was transfected into HEK293T and 293KDR cell lines using standard calcium phosphate method. As expected, the recombinant phagemid could be expressed in both cell lines, exhibiting a variety of fluorescent phenotypes. The percentage of positive cells in HEK293T and 293KDR cells were approximately 20% and 30%, respectively (Figure 2).

***Confirmation of the VEGFR2-specific nanobody expressing phages***

**Figure 3:**
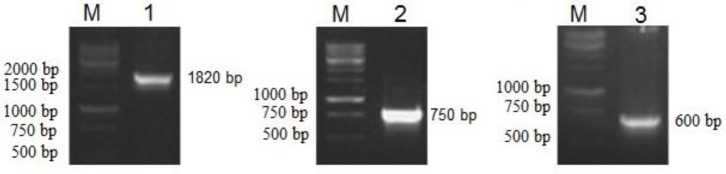
Colony-PCR for verification of resultant recombinant phages. Lanes M, 1-kb molecular weight marker; lane 1, colony-PCR with specific primers pCMV (*EcoR I*) and BGH (*Nar I*), representing ~1820 bp; lane 2, colony-PCR with Cop-GFP (*Nhe I*) and Cop-GFP (*Sac I*) primers, representing ~750 bp; and lane 3, colony-PCR with GIII and Rp primers, representing ~600bp

After obtaining the recombinant phages, PCR was applied to confirm the presence of expression construct containing the Cop-GFP gene, CMV promoter and the other elements in the recombinant phage genome. For this purpose, the recombinant phages were transformed into *E. coli* TG1 and PCR was performed using colonies grown on selective medium. All constructed genes found in the phage genome and confirmed by primers (Figure 3).

***In vitro*** ***transduction*** ***and reporter gene expression ***

To determine if VEGFR2-specific nanobody expressing phages particles are capable of targeting VEGFR2 expressing cell line, the 293KDR cells were incubated with the recombinant phagemids at 37 ^°^C. Subsequently, cells were analyzed for Cop-GFP expression by fluorescence microscopy after 72 hr. Approximately 1% of the 293KDR in our highest concentration of phage (10^11^ cfu/ml) cells showed Cop-GFP expression after incubation with recombinant phagemid. In addition, 293KDR cells incubated with no phage or helper phage failed to exhibit Cop-GFP expression. These data confirmed that phage-mediated gene delivery is restricted to the VEGFR2 nanobody expressing on the surface of phage particles, which is required for VEGFR2-dependent binding and subsequent internalization. 

## Discussion

Phage-mediated gene transfer provides an alternative method of gene delivery into specific cell types. Bacteriophages such as filamentous bacteriophages can be easily engineered to transfer genes into specific types of mammalian cells by attaching a targeting ligand to the phage surface with genetic engineering (14) or non-covalently methods (15). The first report of using the phage in tumor therapy by Bolch have been showed accumulation of phages in tumor will eventually lead to inhibition of tumor growth (16). Following Bolch observation, several *in vivo* and *in vitro* studies have performed to demonstrate the ability of bacteriophages to target cancer cells. For example, in a study T4 bacteriophages were administrated to mice bearing melanoma and migration of melanoma cells on fibronectin was inhibited (17). They have shown that phages are able to bind to melanoma cells, but this interaction is very weak (17). They later discovered that a mutation in *hoc* gene can increase the affinity of the phage for melanoma cells (18, 19). In another study, showed that fifty percent of B16-F10 melanoma tumor bearing mice showed tumor regression by a phage that had tumor targeting peptide on their capsid (20).

Although phages are considered as a safe system for gene delivery into mammalian cells, but the low efficacy of this system in transduction is still a problem. In order for a phage to be successful at transducing a cell, different cellular processes such as vector trafficking, strand conversion and vector copy number can take part. An approach that have successfully applied to increase the efficacy of phage is using multivalent ligand pattern on the surface of phages (21, 22). Transduction levels as high as 10% were obtained in human prostate carcinoma cells transfected with multivalent phagemid vectors (8, 12). This increase can be explained by the fact that increase in the avidity as well as availability of ligand can help the dimerization of cell surface receptor (6). It has also been shown that addition of hydroxycamptothecin (HCPT) can increase the efficacy of transduction of mammalian cell by phagemid (23). In a study by Cai *et al.* for examples, phage particle displaying EGF ligand on their surface and with SiRNA against FAK gene was cloned into phagemid vector was developed and the efficacy of this system was evaluated in HI299. They showed that colony formation and cell invasion of HCPT treated group was less than of the other groups (24). In Larocca *et al*. study, it was observed that the same phage had different transduction efficacy on different cells (6). In other study, phage-mediated transduction efficiency was found to be 1–4% with targeted phages (14). 

The low transduction efficacy which was observed in our study might be overcome by using HCPT or using multivalent ligand approach. Additionally, different types of cells can be test to see whether the low efficacy of transduction of our phage was due to our 293KDR cell line properties. In line with our results, Poul and Marks showed filamentous phage vectors displaying anti-ErbB2 scFv F5 as a genetic fusion with the M13-phage pIII protein can directly infect mammalian cells expressing ErbB2, leading to expression of a reporter gene in the phage genome (25). Larocca *et al.* demonstrated that when phages are genetically engineered to display fibroblast growth factor (FGF)-2 ligand, phages acquire the ability to deliver a gene to mammalian cells through the FGF receptor (26). It is hoped that the results from this study will help to find suitable vectors to improve both efficiency and safety of gene delivery for gene therapy in human. Furthermore, this vector can be used in cancer research to deliver therapeutic agents into tumor. It can also be used to target tumors indirectly by targeting the metabolism of tumor by targeting angiogenesis molecules pathways. These results indicate that phage mediated gene therapy is capable of delivering gene into the target in a very cell specific manner.

## Conclusion

In summary, we described the M13 phage vector with an appropriate tropism for mammalian cells by inserting DNA coding of nanobody gene against VEGFR2 fused to PIII protein of phage. Cop-GFP was also inserted into phage genome as a reporter gene. The recombinant phage vector was internalized by VEGFR2-mediated endocytosis and Cop-GFP expression was observed in ~1% of the 293KDR cells.
